# High Mortality in Adults Hospitalized for Active Tuberculosis in a Low HIV Prevalence Setting

**DOI:** 10.1371/journal.pone.0092077

**Published:** 2014-03-18

**Authors:** Grace Lui, Rity Y. K. Wong, Florence Li, May K. P. Lee, Raymond W. M. Lai, Timothy C. M. Li, Joseph K. M. Kam, Nelson Lee

**Affiliations:** 1 Department of Medicine and Therapeutics, Division of Infectious Diseases, Prince of Wales Hospital, Chinese University of Hong Kong, Hong Kong SAR, China; 2 Faculty of Medicine, University of Sheffield, Sheffield, United Kingdom; 3 Department of Microbiology, Prince of Wales Hospital, Chinese University of Hong Kong, Hong Kong SAR, China; 4 Stanley Ho Centre for Emerging Infectious Diseases, Chinese University of Hong Kong, Hong Kong SAR, China; Temple University School of Medicine, United States Of America

## Abstract

**Background:**

This study aims to evaluate the outcomes of adults hospitalized for tuberculosis in a higher-income region with low HIV prevalence.

**Methods:**

A retrospective cohort study was conducted on all adults hospitalized for pulmonary and/or extrapulmonary tuberculosis in an acute-care hospital in Hong Kong during a two-year period. Microscopy and solid-medium culture were routinely performed. The diagnosis of tuberculosis was made by: (1) positive culture of *M. tuberculosis*, (2) positive *M. tuberculosis* PCR result, (3) histology findings of tuberculosis infection, and/or (4) typical clinico-radiological manifestations of tuberculosis which resolved after anti-TB treatment, in the absence of alternative diagnoses. Time to treatment (‘early’, started during initial admission; ‘late’, subsequent periods), reasons for delay, and short- and long-term survival were analyzed.

**Results:**

Altogether 349 patients were studied [median(IQR) age 62(48–77) years; non-HIV immunocompromised conditions 36.7%; HIV/AIDS 2.0%]. 57.9%, 16.3%, and 25.8% had pulmonary, extrapulmonary, and pulmonary-extrapulmonary tuberculosis respectively. 58.2% was smear-negative; 0.6% multidrug-resistant. 43.4% developed hypoxemia. Crude 90-day and 1-year all-cause mortality was 13.8% and 24.1% respectively. 57.6% and 35.8% received ‘early’ and ‘late’ treatment respectively, latter mostly culture-guided [median(IQR) intervals, 5(3–9) vs. 43(25–61) days]. Diagnosis was unknown before death in 6.6%. Smear-negativity, malignancy, chronic lung diseases, and prior exposure to fluoroquinolones (adjusted-OR 10.6, 95%CI 1.3–85.2) delayed diagnosis of tuberculosis. Failure to receive ‘early’ treatment independently predicted higher mortality (Cox-model, adjusted-HR 1.8, 95%CI 1.1–3.0).

**Conclusions:**

Mortality of hospitalized tuberculosis patients is high. Newer approaches incorporating methods for rapid diagnosis and initiation of anti-tuberculous treatment are urgently required to improve outcomes.

## Introduction

The global burden of tuberculosis (TB) remains enormous, and TB continues to be a major cause of mortality worldwide [1], [2]. Although HIV infection is a potent risk factor for development of active TB, 85–90% of TB cases were reported among HIV-negative persons [1], [2]. In 2011, nearly one million HIV-negative persons died from TB [2]. Most studies on outcomes of patients with active TB however, are reported from developing, lower-income countries and regions with high HIV prevalence [3]–[5], and have largely involved younger adults with less severe infections not requiring hospital care [3], [5], [6]. The risk factors and the short- and long-term clinical outcomes of older, non-HIV patients hospitalized for severe TB infection remain unclear, particularly in higher-income regions [7], [8]. Recent available data suggest that such patients may have a much higher risk of death (4–11 times) than those presented to the outpatient settings [6], [9]. However, diagnosis of TB in these hospitalized patients is often difficult due to co-existing medical conditions, ‘atypical’ manifestations and smear-negativity, and consideration of empirical treatment may be abrogated by potential drug toxicities [10]. The impact of delay in diagnosis and treatment initiation on patient outcome had not been fully elucidated in this population [11].

Hong Kong carries an intermediate TB burden with a notification rate of 74–90 per 100,000 population [12], and has a low HIV prevalence of <0.1% [13]. In this study, we aimed to investigate the short- and long-term survival of adults hospitalized for active TB, the risk factors for increased mortality, and reasons for diagnosis and treatment delay. Our results highlight the high morbidity and mortality of TB among hospitalized patients, and that conventional diagnostic and management approach is presently insufficient to prevent adverse outcomes.

## Materials and Methods

### Study population and management of patients

A retrospective cohort study was performed at the Prince of Wales Hospital, a 1300-bed university-affiliated, general acute-care hospital managed by the Hospital Authority of Hong Kong. It serves an urban population of >1.5 million, accounting for >20% of the population in Hong Kong [14]. The Hospital Authority is the main provider of acute medical services in Hong Kong. All adults aged ≥18 years who were admitted to the hospital during a 2-year period (1st January 2008 to 31st December 2009) for pulmonary and/or extra-pulmonary TB were identified and included for study. Patients who were hospitalized for known TB and had been receiving anti-tuberculous (anti-TB) therapy prior to admission were excluded from this analysis. For patients with multiple admissions, the initial episode when patients first presented after symptom onset was studied.

During the study period, in all patients with suspected active TB infections based on compatible symptoms and/or radiological abnormalities, collected respiratory (e.g. sputum, bronchoalveolar lavage) and non-respiratory tract samples (e.g. pleural fluid/tissue, lymph node aspirates) were routinely examined by both fluorescence microscopy followed by Ziehl-Neelsen staining, and mycobacterial cultures using the Löwenstein-Jensen solid medium followed by conventional drug-susceptibility testing [14]. Liquid-medium cultures (using BACTEC MGIT 960 system) and *Mycobacterium tuberculosis* PCR assay (Gen-Probe Amplified Mycobacterium Tuberculosis Direct Test) were performed upon clinicians' requests. Tissue biopsies were performed at accessible sites by the managing physicians when deemed necessary [14]. The diagnosis of TB was made by one or more of the following: (1) positive culture of *M. tuberculosis*, (2) positive *M. tuberculosis* PCR result, (3) histology findings of TB infection (e.g. acid-fast bacilli and caseating granuloma), and (4) typical clinico-radiological manifestations of TB which responded to and resolved after anti-TB treatment, in the absence of alternative diagnoses. Anti-TB treatment, which typically consists of 4 drugs (e.g. isoniazid, rifampicin, pyrazinamide, ethambutol, or aminoglycoside), was prescribed according to local guidelines [12]. TB is a statutory notifiable disease In Hong Kong, and all patients after hospital discharge are being referred to designated TB and Chest out-patient clinics for continuation of directly-observed anti-TB treatment (DOTS) [12].

### Data retrieval

A complete list of patients was generated via the hospital's computerized ‘Hospital Clinical Management System’ and ‘Laboratory Information System’. Patients' electronic and written records were reviewed, and information was recorded systematically using a standardized research tool for TB [14]. Variables studied included: demographics, non-HIV immunocompromised conditions (diabetes mellitus, malignancy, chronic kidney disease, cirrhosis, use of immunosuppressants, and pregnancy), HIV/AIDS, chronic lung diseases and other medical comorbidities; presenting symptoms; site(s) of infection; exposure to fluoroquinolones prior to diagnosis; mycobacterial and histological investigation results; date of initiation of anti-TB treatment; development of hypoxemia requiring supplementary oxygen support, intensive care unit (ICU) admission, duration of hospitalization, and all-cause mortality. Ethical approval was obtained from the Institutional Review Boards of the Chinese University of Hong Kong and the Hospital Authority of Hong Kong. As patient records were anonymized and de-identified prior to analysis, consent was not obtained from individual patients.

### Definitions and data analysis

‘Early’ anti-TB treatment was defined as therapy started during the initial hospital admission when patient first presented after symptom onset. ‘Late’ treatment referred to therapy started in subsequent periods when the diagnosis of TB was confirmed (e.g. by culture). Patients might have received ‘no’ treatment if the diagnosis was unknown before death, but later confirmed by culture. The main outcome measures were 90-day (short-term) and 1-year (longer-term) all-cause mortality, counting from the date of first hospital admission.

In the univariate analyses, categorical variables were compared using Chi-square or Fisher's exact test, and continuous variables with the Mann Whitney U test. Variables with p values <0.05 were entered into multivariate logistic regression models (backward, stepwise) to identify factors associated with delay in diagnosis and treatment initiation; adjusted Odds Ratios (OR) and 95% confidence intervals (CI) were reported for each explanatory variable. Cox proportional hazards models (backward, stepwise) were used to analyze independent risk factors associated with mortality. Adjusted Hazard Ratios (HR) and 95% CI were calculated for each explanatory variable. A HR >1 indicated a higher chance of death. Kaplan-Meier curve was constructed to show survival of patients who received ‘early’ and ‘late’ anti-TB treatments. All probabilities were two tailed, and a p-value of <0.05 was considered to indicate statistical significance. Statistical analysis was performed using PASW Statistics software, version 17.0.

## Results

A total of 349 TB patients were studied (Table 1), with median age 62 (IQR 48–77) years; of which 71.9% were male, and 36.7% had one or more non-HIV immunocompromised conditions. HIV prevalence was low (2.0%) in our cohort. Pulmonary, extra-pulmonary, and pulmonary-extrapulmonary TB were diagnosed in 57.9%, 16.3%, and 25.8% respectively. TB pleuritis and TB lymphadenopathy were the predominant extra-pulmonary manifestations. Overall, 58.2% were ‘smear-negative’ TB cases; 90.5% were culture positive (n = 316). Only 2 (0.6%) isolates showed resistance to both isoniazid and rifampicin (‘multidrug-resistant (MDR)’), while 30 (9.5%) showed resistance to one or more first-line anti-tuberculous drug. Bilateral pulmonary involvement was noted in 33.9%; 43.4% developed hypoxemia requiring supplementary oxygen therapy, and 5.7% had required ICU-level care. The crude 90-day and 1-year all-cause mortality rate was 13.8% (n = 48) and 24.1% (n = 84) respectively. Analysis of death cases (n = 84) showed that 81 (96.4%) patients had either died before the diagnosis was made (n = 23, 27.4%) or prior to completion of anti-TB treatment (n = 58, 69.0%).

**Table 1:** Clinical presentations, diagnosis, treatment and outcomes of 349 adults hospitalized for TB.

Variables	N = 349
Age, median (IQR), years	62 (48,77)
Gender, male (%)	251 (71.9)
Co-morbidities (%)	
diabetes mellitus	74 (21.3)
malignancy	45 (12.9)
chronic kidney disease	28 (8.0)
immunosuppressant use	7 (2.0)
HIV infection 1	7 (2.0)
chronic lung diseases	51 (14.7)
immunocompromised conditions, any 1	135 (38.8)
Presence of fever (%)	214 (61.7)
Pulmonary TB alone (%)	202 (57.9)
Extra-pulmonary TB alone (%)	57 (16.3)
Both pulmonary and extra-pulmonary TB (%)	90 (25.8)
Involvement sites of extra-pulmonary TB (%)	
pleura	80 (22.9)
lymphadenopathy	23 (6.6)
central nervous system	11 (3.2)
genitourinary	9 (2.6)
bone and joint	9 (2.6)
Confirmation of diagnosis (%)	
smear positivity	146 (41.8)
culture positivity 2	316 (90.5)
PCR positivity 2	47 (13.5)
histological findings 2	86 (24.6)
typical clinico-radiological manifestations alone	18 (5.2)
Radiographic, bilateral lung involvements (%) 3	105 (33.9)
Radiographic, cavitatory lesions (%) 3	45 (14.5)
Hypoxemia, supplementary oxygen required (%)	145 (43.4)
Intensive care unit admission (%)	20 (5.7)
Treatment initiation (%)	
‘early’ anti-TB treatment	201 (57.6)
‘late’ anti-TB treatment	125 (35.8)
Never received anti-TB treatment before death	23 (6.6)
Acute hospital length-of-stay, median (IQR), days	10 (6, 16)
Total length-of-stay, median (IQR), days 4	15 (8, 26)
Crude 90-day mortality, all-cause (%)	48 (13.8)
Crude 1-year mortality, all-cause (%)	84 (24.1)

1 HIV serology testing was performed in 223 patients; Hong Kong population has an overall HIV-prevalence of <0.1% [^13^]. Immunocompromised conditions (any): diabetes mellitus, malignancy, chronic kidney disease, immunosuppressant use, HIV/AIDS, cirrhosis (n = 1), and pregnancy (n = 2).

2 Liquid-medium culture was performed in parallel to solid-medium culture in 166 (47.6%) patients, and 92 (26.4%) showed positive growth (distribution similar between early and late treatment groups, p = 0.459); PCR for *Mycobacterium tuberculosis* was performed in 85 (24.4%) patients; histological examinations: pleural biopsy 41.9%, lung or endobronchial biopsy 25.6%, other sites 32.6%.

3 Radiographic data available in 310 patients.

4 Total length-of-stay: total duration of hospitalization in both acute and convalescent/chest hospitals.

Analysis on time to treatment initiation showed that 57.6% (n = 201) of cases received ‘early’ (median 5 days, IQR 3–9 days), and 35.8% (n = 125) received ‘late’ (median 43 days, IQR 25–61 days) treatment respectively. Initiation of ‘early’ treatment was based on positive-smear results (46.3%), histological findings (17.4%), or typical clinico-radiological manifestations (29.4%); initiation of ‘late’ treatment was largely (68.8%) based on positive culture results (Table 2). Independent factors associated with delayed diagnosis and failure to receive ‘early’ treatment included older age (OR 1.2 per 10-year increase, 95% CI 1.0–1.4, p = 0.018), underlying malignancy (OR 4.2, 95%CI 1.5–11.9, p = 0.007) or chronic lung diseases (OR 2.9, 95%CI 1.1–8.0, p = 0.041), absence of fever (OR 2.9, 95%CI 1.5–5.5, p = 0.001), pulmonary manifestations alone (OR 3.3, 95%CI 1.7–6.7, p = 0.001), smear-negativity (OR 26.6, 95%CI 12.0–58.9, p<0.001), and recent exposure to fluoroquinolones (OR 10.6, 95%CI 1.3–85.2, p = 0.026) (Table S1). The median time to initiate anti-TB was 55 (IQR 24–69) and 9 (IQR 4–31) days in those with or without fluoroquinolone exposure respectively. The use of PCR testing was associated with early diagnosis and initiation of anti-TB treatment.

**Table 2:** Characteristics and outcomes of patients who received early diagnosis and treatment during the initial hospital admission, versus those who were diagnosed late (± treatment).

Variables	Early diagnosis and treatment	Late diagnosis ± treatment		P-values
	N = 201 (%)	N = 148 1 (%)		
Time-to-initiate anti-TB treatment, median(IQR), days 2	5 (3,9)	43 (25,61) 1		<0.001
Age, median (IQR), years	57 (42, 74)	71 (54, 78)		<0.001
Gender, male	137 (68.2)	114 (77.0)		0.068
Co-morbidities				
diabetes mellitus	42 (21.0)	32 (21.6)		0.889
Malignancy	11 (5.5)	34 (23.0)		<0.001
chronic kidney disease	17 (8.5)	11 (7.4)		0.717
HIV infection	6 (3.0)	1 (0.7)		0.246
immunosuppressant use	6 (3.0)	1 (0.7)		0.246
chronic lung diseases	14 (7.0)	37 (25.0)		<0.001
immunocompromised conditions, any	71 (35.5)	64 (43.2)		0.143
Symptom, absence of fever	58 (29.1)	75 (50.7)		<0.001
Symptom, weight loss	80 (40.6)	38 (26.6)		0.007
Symptom, night sweats	41 (21.0)	15 (10.7)		0.013
Pulmonary manifestations alone	107 (53.2)	95 (64.2)		0.041
Radiographic, cavitatory lesions	34 (18.7)	11 (8.6)		0.013
Liquid-medium culture performed	102 (50.7)	64 (43.2)		0.165
PCR performed	59 (29.4)	26 (17.6)		0.011
AFB smear-negativity	75 (37.3)	128 (86.5)		<0.001
Exposure to fluoroquinolones 3	2 (1.0)	15 (10.1)		<0.001
Supplementary oxygen requirement	82 (42.3)	63 (45.0)		0.619
Intensive care unit admission	15 (7.5)	5 (3.4)		0.105
90-day mortality, all-cause	19 (9.5)	29 (19.6)		0.007
1-year mortality, all-cause	33 (16.4)	51 (34.5)		<0.001

1 Altogether, 125 patients received ‘late’ treatment and 23 remained undiagnosed before death thus received no anti-TB treatment (see text). Comparisons between those who had received ‘early’ treatment and those who died before TB diagnosis were described in Table S3.

2 Basis for initiating ‘early’ anti-TB treatment (n = 201): positive-smear 93 (46.3%), positive PCR 8 (4.0%), positive culture 6 (3.0%), histological findings 35 (17.4%), typical clinico-radiological manifestations 59 (29.4%); ‘late’ treatment (n = 125): positive-smear 10 (8.0%), positive PCR 6 (4.8%), positive culture 86 (68.8%), histological findings 12 (9.6%), clinico-radiological manifestations 11 (8.8%).

3 Received fluoroquinolones prior to the diagnosis of TB. Levofloxacin was used in 15 cases, and ciprofloxacin in 2 cases.

**Figure 1:**
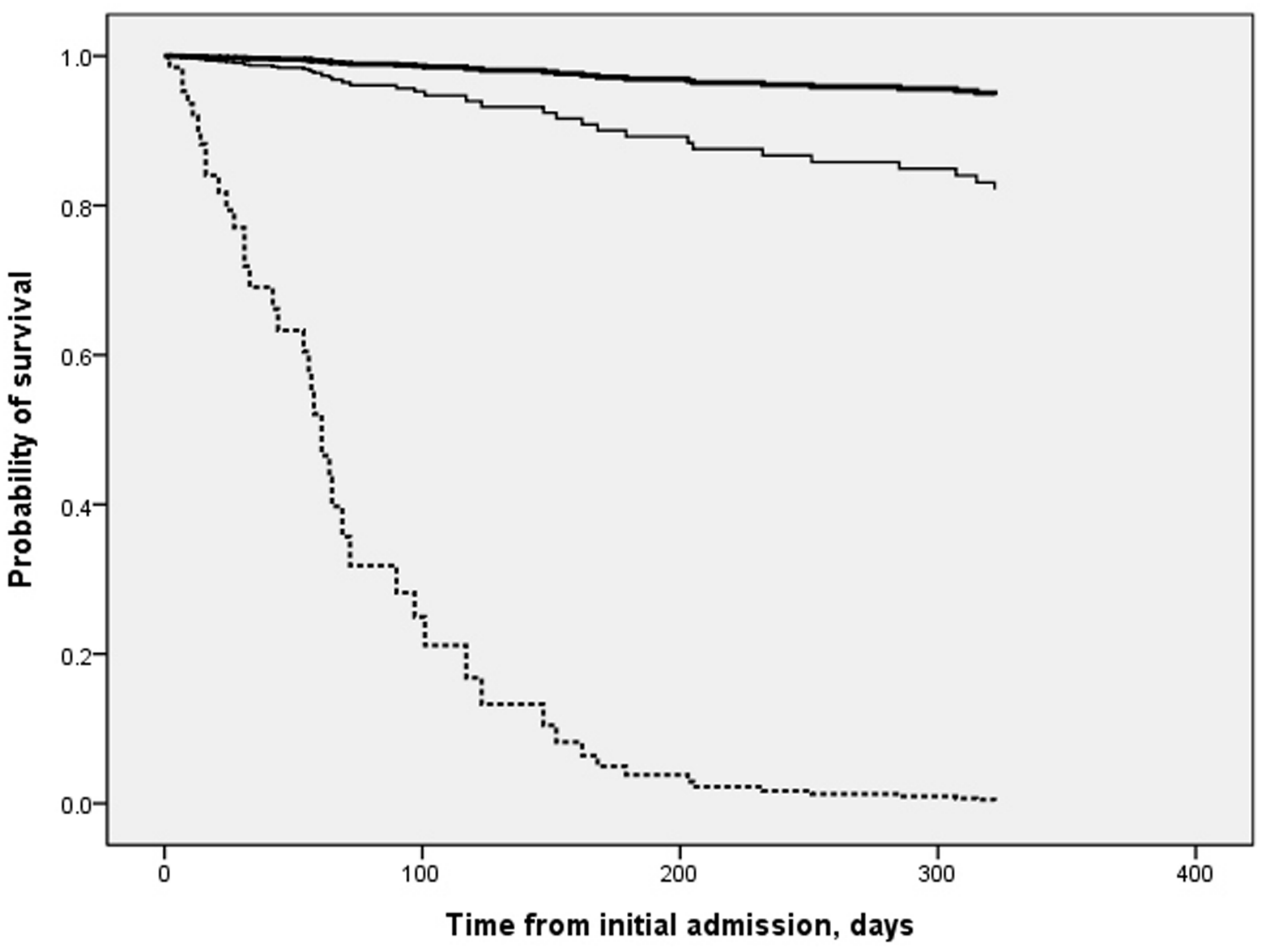
Survival of patients with smear-negative tuberculosis (n = 203), according to time of initiation of anti-TB treatment. Patients who received late (thin solid line)(HR 3.65, 95%CI 1.07–12.40; p = 0.039) or no anti-TB treatment (dotted line)(HR 104.22, 95%CI 24.59–441.66; p<0.001) were shown to have significantly lower survival than those who received early treatment (thick solid line)(reference), as shown in the final Cox proportional hazards model, adjusted for demographics, immunocompromised conditions (HR 3.95, 95%CI 1.85–8.44; p<0.001) and supplemental oxygen requirement (HR 1.98, 95%CI 1.02–3.85; p = 0.043). Basis for initiating ‘early’ anti-TB treatment (n = 75): positive PCR 5 (6.7%), positive culture 5 (6.7%; liquid-medium, 3), histological findings 22 (29.3%), typical clinico-radiological manifestations 43 (57.3%).

For the whole cohort (n = 349), it was shown that in addition to advanced age, underlying immunocompromised conditions and hypoxemia, failure to receive ‘early’ anti-TB treatment was an independent factor associated with higher risk of death at 90 days (HR 2.0, 95%CI 1.1–3.8, p = 0.032) and at 1 year (HR 1.8, 95%CI 1.1–3.0, p = 0.015)(Table 3 and Table S2). The analyses were adjusted for confounders including gender, extra-pulmonary manifestations, smear-negativity, and requirement of ICU care. Subgroup analysis of smear-negative TB cases (n = 203) showed that patients who received late treatment had significantly higher risk of death than those who were treated early, adjusted for potential confounders (HR 3.7, 95%CI 1.1–12.4, p = 0.039; Figure 1 and Table S2).

**Table 3:** Explanatory variables in the final Cox proportional hazards models associated with increased all-cause mortality, events censored at 90 days and 1 year from initial admission (N = 349).

Explanatory variables	All-cause death at 90 days			All-cause death at 1 year		
	Adjusted HR	95% CI	P-value	Adjusted HR	95% CI	P-value
Age, median (IQR)	1.30*	1.05, 1.61	0.016	1.40*	1.18, 1.66	<0.001
Immunocompromised conditions	3.35	1.76, 6.39	<0.001	3.61	2.20, 5.92	<0.001
Supplementary oxygen requirement	2.78	1.39, 5.59	0.004	2.39	1.43, 3.99	0.001
Failure to receive early treatment during initial admission	2.01	1.06, 3.80	0.032	1.83	1.13, 2.96	0.015

*Per 10-year increase in age.

Other covariates in the models included gender, extra-pulmonary manifestations, smear-negativity, and requirement of ICU care.

## Discussion

Our results showed that despite the availability of effective anti-TB treatment, mortality of adult patients hospitalized with active TB had remained substantial even in settings with low HIV prevalence. Diagnosis was often difficult, leading to treatment delay or even omission in >40% of cases. Failure to receive timely treatment was associated with a doubled death risk. Newer approaches to diagnosis and treatment in this unique population are urgently required.

Our observations on mortality (from an intermediate TB burden area) are generally consistent with available reports from other higher-income regions with low HIV prevalence, which showed in-hospital and short-term death rates exceeding 10% [7], [11], [15], and longer-term mortality about 20-30% [6], [16], [17]. This reflects the complicated and often protracted courses of illness in such patients despite started on anti-TB treatment. Nearly all deaths occurred prior to completion of the intended treatment course (∼69%), or before the diagnosis could be made (∼27%) [16], [18]. We identified several risk factors for increased mortality in this cohort which included advanced age, underlying non-HIV immunocompromised conditions such as diabetes mellitus [15], chronic kidney disease [6], [15]–[17], malignancy [6], [15], [16], and use of immunosuppressants, and development of respiratory failure during the course of illness [7], [17]. Extra-pulmonary manifestations (70% were pleuritis or lymphadenitis in our cohort) and AFB smear-positivity/negativity alone were not shown to be associated with increased death risks.

Importantly, our analyses suggest that early diagnosis and initiation of anti-TB treatment may reduce mortality in non-HIV adults hospitalized for TB. The unadjusted death rates of patients who received diagnosis and treatment at the time of initial admission was about half of those who did not (mortality at 90 days, 9.5% vs. 19.6%; at 1 year, 16.4% vs. 34.5%; both p<0.01). The association between early treatment and lower mortality remained significant even after adjustment for patient characteristics and disease severity (Table 3). Subgroup analysis of smear-negative TB cases further indicated that patients who received later treatment in a few weeks' time, mostly guided by conventional cultures, had significantly lower survival than those who received early treatment guided by other means (Figure 1) [11], [19]–[21]. It should also be pointed that delayed diagnosis, including the smear-negative TB cases, would result in nosocomial transmission to both patients and healthcare workers especially for MDR cases [22]–[24]. For these reasons, every effort should be made to diagnose and treat TB patients at the earliest opportunity.

However, diagnosis of TB in this patient group has remained difficult. Underlying lung diseases or malignancies [25], [26], and ‘atypical’ manifestations such as absence of fever or non-cavitatory radiographic lesions may confuse the diagnosis [18], [21], [27]. Further, a large proportion of TB cases (nearly 60% in our cohort) may be smear-negative, thus cannot be promptly diagnosed without the use of more advanced methods [1], [11], [21], [25]. Use of PCR testing aided early diagnosis in our study. The high specificity of the currently available commercial nucleic acid amplification tests was shown to allow “ruling in” TB by a couple of weeks earlier than culture in smear-negative TB [28]. Newer, more sensitive methods, including automated liquid-medium or other rapid culture systems (e.g. microscopically observed drug susceptibility) [1], real-time PCR (e.g. XPERT MTB/RIF assay) [29], and antigen detection assays (e.g. urine lipoarabinomannan) [1] have shown promise and can be considered if available. Further evaluation of their performance and cost-effectiveness, either single or in combination as in a diagnostic algorithm, is urgently required in the hospitalized patients [29]. If unavailable, tissue biopsy (particularly at the more accessible sites, e.g. pleura, lymph node) [30] and empirical anti-TB treatment, especially in patients at high risk for mortality, should be considered with careful monitoring of drug-related toxicity and review of culture results [31], [32]. Future studies should be conducted to explore clinical practices and attitudes about empiric TB treatment in hospitalized patients. Fluoroquinolones can partially suppress clinico-radiological manifestations of TB [33], [34] and decrease diagnostic yield of mycobacterial assays [35], leading to delayed treatment and mortality [25], [33], [34], [36]. As such, fluoroquinolones should be used cautiously in the treatment of patients with acute bacterial pneumonia patients in TB endemic areas, and further investigations and close follow up should be arranged for those who had received fluoroquinolones.

Our study has several limitations. First, the study was limited to patients hospitalized at a single hospital and patients diagnosed in the outpatient setting of the catchment area were not included, thus limiting its generalizability. There is no current consensus in defining ‘early’ and ‘late’ treatment [37], thus we have taken a pragmatic approach to classify those started during the first admission as ‘early’ (nearly all within 10 days), and those given later because of initial misdiagnosis as ‘late’ treatment (median delay about 6 weeks). Such classification thus emphasized the impact of missed opportunity for starting treatment if relied on conventional diagnostic means. The possible impact of patient delay could not be analyzed because retrospective data on time of onset of TB symptoms was imprecise; available recorded data indicated that time-to-presentation was within 2–3 weeks in both groups. Confounders such as old age, immunocompromised conditions and severity had been carefully adjusted in multivariate models when examining the effect of treatment delay on outcomes. Finally, the lack of standard use of a liquid-medium culture system is a limitation of this analysis.

In conclusion, we found that non-HIV adults hospitalized for TB have high mortality, which can be related to host factors, disease severity, and treatment delay. Given the complexity of the medical scenarios in such patients, newer approaches for rapid diagnosis and a validated diagnostic algorithm to guide treatment are urgently required to improve outcomes.

## Supporting Information

Table S1 **Explanatory variables in the final logistic regression model analyzing factors associated with failure to receive early diagnosis and treatment in 349 hospitalized TB patients.**
(DOCX)

Table S2 **Comparisons of patients who survived or died within (a) 90 days and (b) 1 year from initial hospital admission, whole cohort; (c) smear-negative TB cases only.**
(DOCX)

Table S3 **Characteristics and outcomes of patients who received early diagnosis and treatment during the initial hospital admission, versus those who died before TB diagnosis.**
(DOCX)
