# Supplementary material for: High Mortality in Adults Hospitalized for Active Tuberculosis in a Low HIV Prevalence Setting
Source: PLoS One. 2014 Mar 18;9(3):e92077. doi: 10.1371/journal.pone.0092077 (PMC3958438; doi:10.1371/journal.pone.0092077)
Supplement: Table S1 — Explanatory variables in the final logistic regression model analyzing factors associated with failure to receive early diagnosis and treatment in 349 hospitalized TB patients. (DOCX) [file pone.0092077.s001.docx]

**Table S1. Explanatory variables in the final logistic regression model analyzing factors associated with failure to receive early diagnosis and treatment in 349 hospitalized TB patients**

| **Variables** | **Adjusted OR** | **95% CI** | **P-values** |
| --- | --- | --- | --- |
| age, median (IQR), years | 1.22* | 1.04, 1.44 | 0.018 |
| malignancy | 4.20 | 1.49, 11.88 | 0.007 |
| chronic lung diseases | 2.88 | 1.05, 7.95 | 0.041 |
| symptom, absence of fever | 2.88 | 1.50, 5.54 | 0.001 |
| pulmonary manifestations alone | 3.33 | 1.65, 6.72 | 0.001 |
| AFB smear-negativity | 26.59 | 12.01, 58.88 | <0.001 |
| exposure to fluoroquinolones | 10.64 | 1.33, 85.22 | 0.026 |

* Per 10 year increase in age

Other covariates included in the final model: gender, symptoms of weight loss and night sweats, and radiographic cavitatory lesions.
