# Supplementary material for: High Mortality in Adults Hospitalized for Active Tuberculosis in a Low HIV Prevalence Setting
Source: PLoS One. 2014 Mar 18;9(3):e92077. doi: 10.1371/journal.pone.0092077 (PMC3958438; doi:10.1371/journal.pone.0092077)
Supplement: Table S2 — Comparisons of patients who survived or died within (a) 90 days and (b) 1 year from initial hospital admission, whole cohort; (c) smear-negative TB cases only. (DOCX) [file pone.0092077.s002.docx]

**Table S2. Comparisons of patients who survived or died within (a) 90 days and (b) 1 year from initial hospital admission, whole cohort; (c) smear-negative TB cases only**

**(a)**

| **Variables** | **Survive at 90 days** | **Died at 90 days** | **P-values** |
| --- | --- | --- | --- |
|  | **N=301 (%)** | **N=48 (%)** |  |
| Age, median (IQR), years | 59 (45, 75) | 77 (68, 85) | <0.001 |
| Gender, male | 213 (70.8) | 38 (79.2) | 0.229 |
| Immunocompromised conditions | 103 (34.2) | 32 (68.1) | <0.001 |
| Presence of fever | 188 (62.5) | 26 (56.5) | 0.441 |
| Extrapulmonary manifestations | 124 (41.2) | 23 (47.9) | 0.381 |
| AFB smear-positivity | 130 (43.2) | 16 (33.3) | 0.199 |
| Supplementary oxygen requirement | 113 (39.0) | 32 (72.7) | <0.001 |
| Intensive care unit admission | 13 (4.3) | 7 (14.6) | 0.011 |
| Failure to receive early treatment during initial admission | 119 (39.5) | 29 (60.4) | 0.007 |

**(b)**

| **Variables** | **Survive at 1 year** | **Died at 1 year** | **P-values** |
| --- | --- | --- | --- |
|  | **N=265 (%)** | **N=84 (%)** |  |
| Age, median (IQR), years | 57 (43, 73) | 76.5 (68, 84) | <0.001 |
| Gender, male | 181 (68.3) | 70 (83.3) | 0.008 |
| Immunocompromised conditions | 77 (29.1) | 58 (69.9) | <0.001 |
| Presence of fever | 164 (61.9) | 50 (61.0) | 0.882 |
| Extrapulmonary manifestations | 114 (43.0) | 33 (39.3) | 0.546 |
| AFB smear-positivity | 114 (43.0) | 32 (38.1) | 0.425 |
| Supplementary oxygen requirement | 94 (36.2) | 51 (68.9) | <0.001 |
| Intensive care unit admission | 11 (4.2) | 9 (10.7) | 0.032 |
| Failure to receive early treatment during initial admission | 97 (36.6) | 51 (60.7) | <0.001 |

**(c)**

| **Variables** | **Survive at 1 year** | **Died at 1 year** | **P-values** |
| --- | --- | --- | --- |
|  | **N=151 (%)** | **N=52 (%)** |  |
| Age, median (IQR), years | 59 (42,75) | 77 (68,86) | <0.001 |
| Gender, male | 101 (66.9) | 43 (82.7) | 0.030 |
| Immunocompromised conditions | 34 (22.5) | 39 (76.5) | <0.001 |
| Presence of fever | 91 (60.3) | 33 (66.0) | 0.470 |
| Extrapulmonary manifestations | 80 (53.0) | 23 (44.2) | 0.276 |
| Supplementary oxygen requirement | 51 (34.7) | 31 (68.9) | <0.001 |
| Intensive care unit admission | 6 (4.0) | 5 (9.6) | 0.154 |
| ‘Late’ treatment | 83 (55.0) | 22 (42.3) | 0.037 |
| ‘No’ treatment | 0 (0.0) | 23 (44.2) | <0.001 |
