# Supplementary material for: High Mortality in Adults Hospitalized for Active Tuberculosis in a Low HIV Prevalence Setting
Source: PLoS One. 2014 Mar 18;9(3):e92077. doi: 10.1371/journal.pone.0092077 (PMC3958438; doi:10.1371/journal.pone.0092077)
Supplement: Table S3 — Characteristics and outcomes of patients who received early diagnosis and treatment during the initial hospital admission, versus those who died before TB diagnosis. (DOCX) [file pone.0092077.s003.docx]

**Table S3. Characteristics and outcomes of patients who received early diagnosis and treatment during the initial hospital admission, versus those who died before TB diagnosis**

| **Variables** | **Early diagnosis  and treatment** | **Death before TB diagnosis** | | **P-values** |
| --- | --- | --- | --- | --- |
|  | **N=201 (%)** | **N=23 (%)** | |  |
| Age, median (IQR), years | 57 (42, 74) | 78 (68, 87) | | <0.001 |
| Gender, male | 137 (68.2) | 18 (78.3) | | 0.320 |
| Co-morbidities |  |  |  |  |
| diabetes mellitus | 42 (21.0) | 6 (26.1) | | 0.594 |
| Malignancy | 11 (5.5) | 13 (56.5) | | <0.001 |
| chronic kidney disease | 17 (8.5) | 2 (8.7) | | 1.000 |
| HIV infection | 6 (3.0) | 0 (0.0) | | 1.000 |
| immunosuppressant use | 6 (3.0) | 0 (0.0) | | 1.000 |
| chronic lung diseases | 14 (7.0) | 7 (30.4) | | 0.002 |
| immunocompromised conditions, any | 71 (35.5) | 18 (78.3) | | <0.001 |
| Symptom, absence of fever | 58 (29.1) | 11 (47.8) | | 0.067 |
| Symptom, weight loss | 80 (40.6) | 4 (19.0) | | 0.054 |
| Symptom, night sweats | 41 (21.0) | 0 (0.0) | | 0.016 |
| Pulmonary manifestations alone | 107 (53.2) | 12 (52.2) | | 0.923 |
| Radiographic, cavitatory lesions | 34 (18.7) | 1 (5.0) | | 0.210 |
| Liquid-medium culture performed | 102 (50.7%) | 11 (47.8%) | | 0.791 |
| PCR performed | 59 (29.4%) | 1 (4.3%) | | 0.010 |
| AFB smear-negativity | 75 (37.3) | 23 (100.0) | | <0.001 |
| Exposure to fluoroquinolones **^3^** | 2 (1.0) | 1 (4.3) | | 0.279 |
| Supplementary oxygen requirement | 82 (42.3) | 17 (73.9) | | 0.004 |
| Intensive care unit admission | 15 (7.5) | 2 (8.7) | | 0.689 |
| 90-day mortality, all-cause | 19 (9.5) | 22 (95.7) | | <0.001 |
| 1-year mortality, all-cause | 33 (16.4) | 23 (100.0) | | <0.001 |
